# *Labisia pumila* Upregulates Peroxisome Proliferator-Activated Receptor Gamma Expression in Rat Adipose Tissues and 3T3-L1 Adipocytes

**DOI:** 10.1155/2013/808914

**Published:** 2013-07-07

**Authors:** Fazliana Mansor, Harvest F. Gu, Claes-Göran Östenson, Louise Mannerås-Holm, Elisabet Stener-Victorin, Wan Nazaimoon Wan Mohamud

**Affiliations:** ^1^Cardiovascular, Diabetes and Nutrition Research Centre, Institute for Medical Research, 50588 Jalan Pahang, Kuala Lumpur, Malaysia; ^2^Department of Molecular Medicine and Surgery, Karolinska Institute, 171 76 Stockholm, Sweden; ^3^Department of Physiology, Institute of Neuroscience and Physiology, University of Gothenburg, Sahlgrenska Academy, 405 30 Gothenburg, Sweden

## Abstract

Peroxisome proliferator-activated receptor gamma (PPARgamma) is a ligand-activated transcription factor that regulates lipid and glucose metabolism. We investigated the effects of *Labisia pumila* (LP) standardized water extract on PPARgamma transcriptional activity in adipocytes *in vitro* and *in vivo*. We used a rat model of dihydrotestosterone- (DHT-) induced polycystic ovary syndrome (PCOS), a condition characterized by insulin resistance. At 9 weeks of age, the PCOS rats were randomly subdivided into two groups: PCOS-LP (50 mg/kg/day of LP) and PCOS-control (1 mL of deionised water) for 4-5 weeks on the same schedule. Real-time RT-PCR was performed to determine the PPARgamma mRNA levels. LP upregulated PPARgamma mRNA level by 40% in the PCOS rats. Western blot analysis further demonstrated the increased PPARgamma protein levels in parallel with upregulation in mRNA. These observations were further proven by adipocytes culture. Differentiated 3T3-L1 adipocytes were treated with final concentration of 100 **μ**g/mL LP and compared to untreated control and 10 **μ**M of rosiglitazone (in type of thiazolidinediones). LP increased PPARgamma expressions at both mRNA and protein levels and enhanced the effect of glucose uptake in the insulin-resistant cells. The data suggest that LP may ameliorate insulin resistance in adipocytes via the upregulation of PPARgamma pathway.

## 1. Introduction 

*Labisia pumila *var. *alata *(LP) (family, Myrsinaceae) or its local name, Kacip Fatimah, is a herbal plant with long history of being used as traditional medicine by Asian women especially those from the Malay Archipelago [1]. The plant grows in lowland primary forest at shady places or in secondary forests on humus-rich soils [2]. The water decoction is traditionally consumed to maintain women's pre- and postpartum health [1, 3]. Components identified in LP extracts include flavonoids, ascorbicacid, beta-carotene, anthocyanin, phenols, and total saponins [4, 5]. 

Insulin resistance is characterized by a decrease in the uptake of glucose, especially by insulin target tissues, including adipose tissue and skeletal muscle [6]. Instead of merely being a storage depot, adipose tissue is now recognized as an important regulator of energy homeostasis [7] and a central player in the development of insulin resistance [8, 9]. One key factor in supporting the central role of adipose tissue in whole-body glucose metabolism is peroxisome proliferator-activated receptor gamma (PPARgamma), a nuclear receptor that is critical both for adipocyte differentiation and for maintenance of mature adipocytes. PPARgamma has the highest expression levels in adipose tissue compared with other metabolic organs, such as skeletal muscle, liver, and pancreas. It can be activated by synthetic compounds, including thiazolidinediones (TZDs) which are used clinically as insulin-sensitizing drugs and antidiabetic agents [10, 11]. A representative of TZD, rosiglitazone, is a potent agonist of PPARgamma and improves the differentiation of 3T3-L1 cells into adipocytes [12, 13]. Treatment with PPARgamma agonists ameliorates insulin sensitivity along with the increase in the plasma adiponectin levels in rodents and human subjects, which at least in part contributes to its ameliorative effect on insulin resistance [14–16]. Some dietary compounds such as dietary lipids, isoflavones, and other flavonoids bind and transactivate PPARgamma [17]. 

Polycystic ovary syndrome (PCOS) is one of the most common endocrine and metabolic disorders, affecting approximately 5–10% of women in reproductive age. It is a complex endocrine and metabolic disorder associated with ovulatory dysfunction, hyperandrogenism, polycystic ovaries, insulin resistance, abdominal fat, and obesity [18–22]. In this study we determine the effects of LP on PPARgamma expressions in the adipose tissues of DHT-induced PCOS rats and glucose uptake in 3T3-L1 adipocytes. 

## 2. Materials and Methods 

### 2.1. Preparation of Standardized *Labisia pumila *Aqueous Extract

Preparation of aqueous LP extract was as described earlier [23]. The plant materials were obtained from central Peninsular Malaysia and a voucher specimen (FRI54816) was deposited at the Herbarium of FRIM. The standardized LPva aqueous extract was produced by a certified Good Manufacturing Practices herbal manufacturing facility using the method described in patent document number US 7879368 B2 [24]. The marker compound used was 3,4,5-trihydroxybenzoic acid and typical yield was between 4 and 5%. The leaves of LP were first oven-dried at 40°C for 3 days. Briefly, the method involved slow heating of the dried leaves in water at 80°C over a period of 3 h, with continuous stirring. This extraction was repeated with an equal volume of fresh water, that is, a two-stage process, in the ratio of one part dried plant material to six parts of water. A spray-drying method was used to concentrate the extract to dryness, in which the tower inlet and outlet temperatures were set at 185 and 107°C, respectively. The spray-dried extract was kept at 4–8°C, in a dessicator, away from light and only sufficient amount was taken each time for use. 

### 2.2. Animal Care and Treatments

Wistar rats were continuously exposed to dihydrotestosterone (DHT) from puberty to adult age to develop PCOS characteristics as previously described [25]. After 7 wk of DHT exposure, rats were randomized into two groups, PCOS-LP (*n* = 11) and PCOS-control (*n* = 11).

The PCOS-LP group received extracted LP orally (50 mg/kg of body weight dissolved in 1 mL of distilled water) daily during 4-5 weeks; the PCOS-control group received 1 mL of distilled water. The dose of LPva was based on previous work by our group [26] and we decided to use the maximum dose applied which influenced several biochemical and physiological variables in these studies. At the end of the experiment, mesenteric adipose tissue was harvested and kept in RNA later stabilization solution (Ambion, Austin, TX, USA) for 24 h at 4°C and then stored at −80°C for mRNA analyses. The study was approved by the Animal Ethics Committee of the University of Gothenburg. 

### 2.3. Euglycemic Hyperinsulinemic Clamp

At 14-15 weeks of age (i.e., after 4-5 weeks of treatment, 10-11 weeks after pellet implantation), rats were subjected to a euglycemic-hyperinsulinemic clamp. The rats were anesthetized with thiobutabarbital sodium (130 mg/kg i.p.; Inactin, Sigma, St. Louis, MO, USA). Catheters were inserted into the left carotid artery and the right jugular vein. A tracheotomy was performed to facilitate respiration. Body temperature was maintained at 37°C with a heating pad. Insulin (100 IU/mL; Actrapid, Novo Nordisk, Bagsvaerd, Denmark) together with 0.2 mL of albumin and 10 mL of physiological saline was infused at 24, 16, and 12 mU/min/kg for 1, 2, and 3 min, respectively, followed by 8 mU/min/kg for the rest of the clamp. Simultaneously, a 20% glucose solution in physiological saline was administered to maintain blood glucose levels at a euglycemic level (6.0 mM). The glucose infusion rate was guided by measuring glucose concentration every 5 min with a B-glucose analyser (Hemocue, Dronfield, Derbyshire, UK). The mean glucose infusion rate, normalized to body weight, was calculated at steady state (after approximately 50–70 min) as an index of insulin sensitivity. Blood samples were taken at the end of the clamp to determine insulin concentrations. Due to failure of the clamp, three PCOS control rat and one PCOS LP rats were excluded from the analyses of mRNA and protein expressions. 

### 2.4. Cell Culture and Treatment

Mouse 3T3-L1 (ATCC CL173) preadipocyte cells were maintained in DMEM containing 10% calf serum. For the differentiation, postconfluent 3T3-L1 preadipocytes (referred to as *day 0*) were treated with DMEM containing 10% FBS, 10 *μ*g/mL insulin, 0.5 mM 3-isobutyl-1-methylxanthine, and 1 *μ*M dexamethasone for 2 days and were then treated for 2 days with DMEM containing 10 *μ*g/mL insulin and 10% FBS. Thereafter, cells were maintained and refed every 2 days with DMEM containing 10% FBS. With this protocol, >80% adipocyte differentiation was achieved. 3T3-L1 cells were treated with final concentrations of 100 *μ*g/mL of LP or 10 *μ*M of rosiglitazone, a type of TZD (Sigma-Aldrich), along with the differentiation medium containing INS-DEX-IBMX since day 0. Control cells were treated with the same volume of DMSO. At day 9, adipocytes were incubated in low-glucose DMEM (GIBCO-BRL) containing 2% (wt/vol) fatty acid-free BSA and were serum starved for 24 h. At day 10, the RNA and protein were extracted for further analysis. 

### 2.5. Analysis of mRNA by Quantitative Real-Time PCR

Total RNA was isolated from adipose tissues cells using RNeasy Kit (Qiagen Inc. Germany). The RNA concentration was quantitated by measuring the absorbency at 260 and 280 nm. First-strand cDNA was synthesized from 2 *μ*g of total RNA extracted using TaqMan Reverse Transcription Reagent (Applied Biosystems, Foster City, CA, USA). The resulting cDNA solution was diluted 45 times and stored at −20°C for later use. In the second step, 100 ng cDNA was used for PCR using TaqMan Universal PCR Master Mix (Applied Biosystems, Branchburg, NJ, USA) in a 96-well plate according to the manufacturer's instructions. We used TaqMan specific primers for PPARgamma, and *β*-actin in our experiments was purchased from Applied Biosystems (Warrington, UK). The real-time quantitative PCR and analysis were carried out using the ABI Prism 7300 Real-time PCR System (Foster City, CA, USA). The relative quantification was determined by the standard curve method for both target and endogenous reference. Real-time PCR analyses were run in triplicate and expression was normalized to the levels of the housekeeping control *β*-actin. 

### 2.6. Protein Expression Levels

#### 2.6.1. Preparation of Adipose Tissue Extract

Frozen tissues were homogenized in a buffer containing 150 mmol/L NaCl, 10 mmol/L Tris, pH 7.4, 1 mmol/L (ethylenebis (oxyethylenenitrilo)) tetraacetic acid, 1 mmol/L EDTA, 1% Triton-X 100, 0.5% Igepal CA-630, 1 *μ*mol/L phenylmethylsulphonyl fluoride, 1 *μ*mol/L pepstatin, 50 trypsin inhibitory milliunits of aprotinin, 10 *μ*mol/L leupeptin, and 2 mmol/L sodium vanadate). Homogenates were centrifuged for 10 min at 9,000 g to remove any debris and insoluble material and then analysed for protein content using BCA protein assay (Pierce Chemical Co., Rockland, IL, USA).

#### 2.6.2. Preparation of Whole Cell Extracts

Monolayers of 3T3-L1 adipocytes were rinsed with phosphate-buffered saline and then harvested in a nondenaturing buffer containing 150 mmol/L NaCl, 10 mmol/L Tris, pH 7.4, 1 mmol/L (ethylenebis (oxyethylenenitrilo)) tetraacetic acid, 1 mmol/L EDTA, 1% Triton-X 100, 0.5% IGEPAL CA-630 (Nonidet P-40), 1 *μ*mol/L phenylmethylsulfonyl fluoride, 1 *μ*mol/L pepstatin, 50 trypsin inhibitory milliunits of aprotinin, 10 *μ*mol/L leupeptin, and 2 mmol/L sodium vanadate. Samples were extracted on ice for 30 min and centrifuged at 13,000 g at 4°C for 10 min. Supernatants containing whole cell extracts were analysed for protein concentration using BCA protein assay (Pierce Chemical Co., Rockland, IL, USA) according to the manufacturer's instructions. 

#### 2.6.3. Gel Electrophoresis and Western Blot Analysis

Cell lysates were prepared in buffer containing a 1 : 100 dilution of protease inhibitor cocktail III (Calbiochem, La Jolla, CA, USA), electrophoresed on SDS-polyacrylamide gels. The proteins were transferred to polyvinylidene difluoride membrane by electroblotting and blocked for 1 h in 5% nonfat dry milk. The membrane then incubated for 1 h with polyclonal antibodies against PPARgamma (Abcam, Cambridge, UK) and later probed with horseradish peroxidase-conjugated secondary antibodies (Abcam). Finally, the membrane was washed and developed using SuperSignal West Pico chemiluminescence reagent (Pierce Chemical Co. Rockford, IL, USA). Densitometry was performed using Bio-Rad Laboratories, Inc. molecular analyst software (Hercules, CA, USA). 

### 2.7. Uptake of 2-Deoxyglucose

Uptake of 2-deoxylglucose by the 3T3-L1 adipocytes was measured as previously described [27]. To induce insulin resistance, 3T3-L1 adipocytes were incubated in low glucose DMEM containing 10−8 M insulin for the last 16 h of serum starvation. Cells were treated with or without 100 *μ*g/mL of LP or 10 *μ*M of rosiglitazone throughout the differentiation at 37°C and then stimulated with or without 100 nM insulin for the final 1 h at 37°C. The potency of LP for PPARgamma was estimated according to EC50 value of 96.78 *μ*g/mL (results not shown) Ten *μ*M of rosiglitazone was used in various experiments [28, 29]. Glucose uptake was initiated by the addition of 2-deoxy-d-[3H] glucose at a final concentration of 3 *μ*mol/L for 10 min in HEPES buffer saline (140 mM NaCl, 5 mM KCl, 2.5 mM MgCl_2_, 1 mM CaCl_2_, 20 mM HEPES, and pH 7.4). The reaction was terminated by separating cells from the HEPES buffer saline and 2-deoxy-d-[3H] glucose. After three washes in ice-cold PBS, the cells were extracted with 0.1% SDS and subjected to scintillation counting for 3H radioactivity. The protein concentration was determined with a BCA assay kit (Pierce, Rockford, IL, USA), and the radioactivities were normalized by determining each total protein concentration.

### 2.8. Statistical Analysis

Each experiment was performed in triplicate. All results were expressed as mean ± SEM Comparisons were analyzed by the Mann-Whitney *U* test and 0ne-way ANOVA followed by Newman-Keuls multiple comparison test. Differences were considered statistically significantly if the *P*  value is less than 0.05. 

## 3. Results 

### 3.1. Insulin Sensitivity

**Figure 1:**
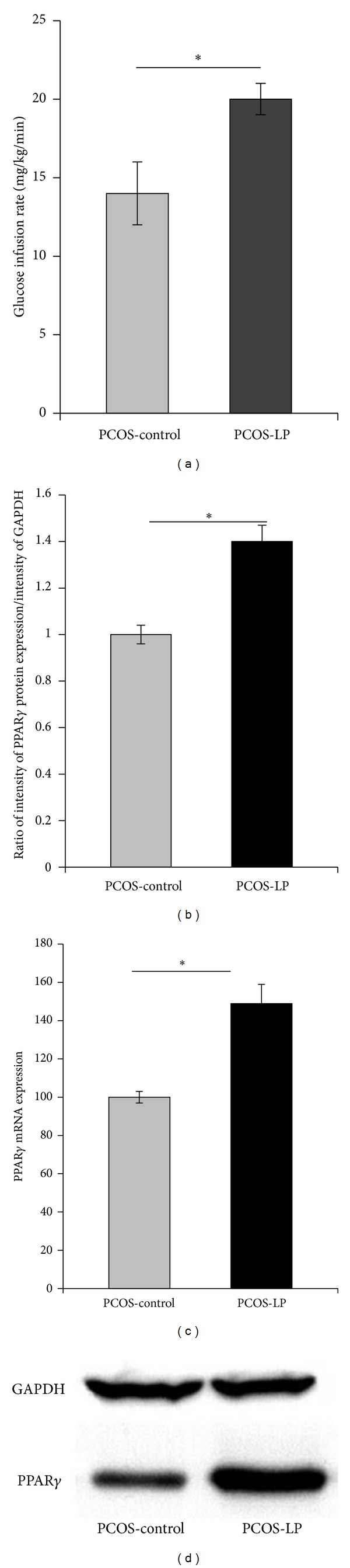
(a) Glucose infusion rate, at steady state, required to maintain euglycemia during euglycemic-hyperinsulinemic clamp in PCOS LPva rats and PCOS controls. Values are mean ± SEM, PCOS LP versus PCOS-control, *P < 0.05 (the Mann-Whitney *U* test). (b) Quantitative blot of PPARgamma protein in PCOS rat adipose tissues. (c) Relative PPARgamma mRNA expression in the adipose tissue of PCOS control (*n* = 8) versus PCOS LP (*n* = 10). The mRNA was corrected by *β*-actin. The level of the expression in the control group was arbitrarily set at 100%. (d) Representative Western blot of PPARgamma in PCOS rat adipose tissues. Western blots of total protein were probed with anti-PPARgamma antibodies and then reprobed with anti-GAPDH to confirm equal loading. Densitometric analyses presented as the relative ratio of PPARgamma to GAPDH. The results represent mean ± S.E.M. from 3 independent experiments. *P < 0.05 as determined by the Mann-Whitney *U* test.

LP-increases insulin sensitivity. The glucose infusion rate, determined by a euglycemic-hyperinsulinemic clamp, was 35% higher in LP treated rats than in controls, indicating improved insulin sensitivity (Figure 1(a)). 

### 3.2. mRNA Levels of the PPARgamma Gene

**Figure 2:**
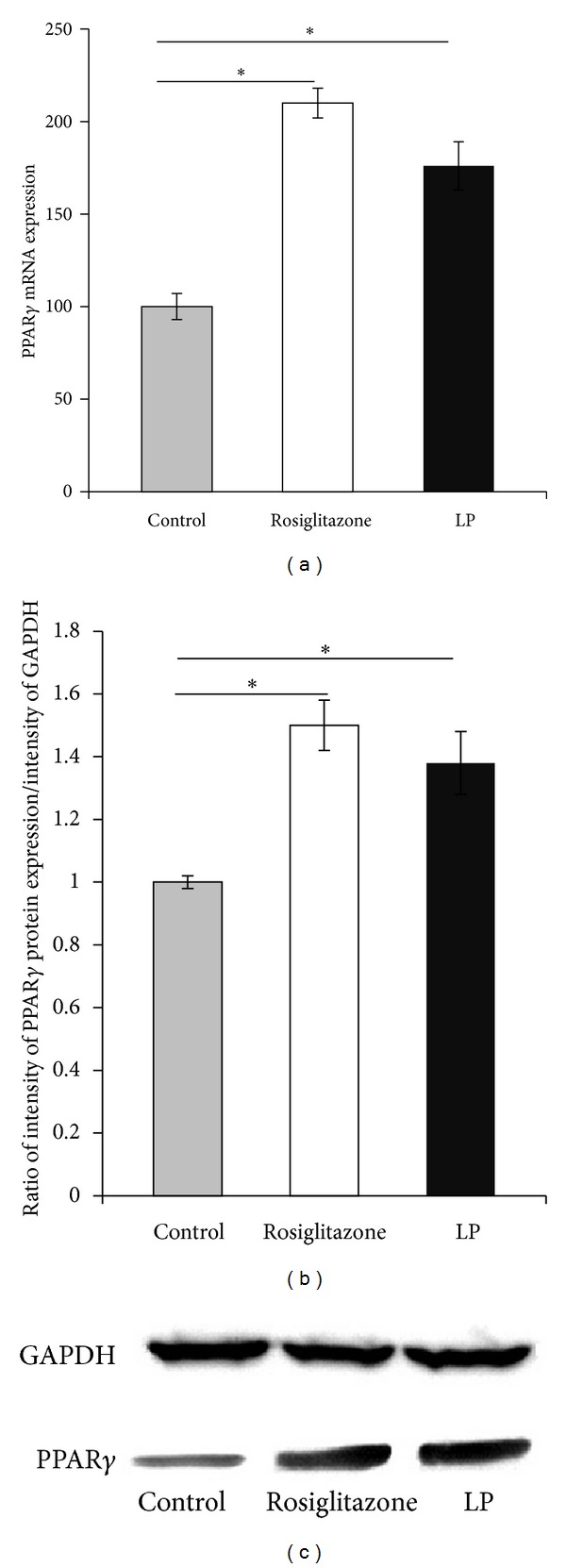
Effect of LP mRNA and protein expressions on3T3-L1 adipocytes. (a) Relative PPARgamma mRNA expression in the 3T3-L1 (*n* = 10). *P < 0.05. The mRNA was corrected by *β*-actin. The level of the expression in the control group was arbitrarily set at 100%. (b) Quantitative blot of PPARgamma protein in 3T3-L1 adipocytes. (c) Representative Western blot of PPARgamma protein in 3T3-L1. Western blots of total protein were probed with anti-PPARgamma antibodies and then reprobed with anti-GAPDH to confirm equal loading. Densitometric analyses presented as the relative ratio of PPARgamma to GAPDH. The results represent mean ± SEM from 3 independent experiments. *P < 0.05 as determined by the Mann-Whitney *U* test.

To determine the PPARgamma mRNA levels in 3T3-L1 and rat adipose tissues, real-time RT-PCR method was used. LP treatment upregulated the PPARgamma expression in mesenteric adipose tissue in DHT-induced PCOS rats (Figure 1(b)). The expression of PPARgamma in 3T3-L1 was markedly increased by TZD treatment, as expected. LP equally up-regulated PPARgamma as TZD (Figure 2(a)). 

### 3.3. Protein Expression Levels of PPARgamma

To determine the translational level of PPARgamma mRNA into protein, the protein levels were assessed by Western blot analysis. The mesenteric PPARgamma protein expression was increased after 4-5 weeks of LP treatment in DHT-induced compared with control PCOS rats (Figures 1(c) and 1(d)). TZD and LP equally increased PPARgamma protein levels in the 3T3-L1 adipocytes (Figures 2(b) and 2(c)). 

### 3.4. Effects of Glucose Uptake by LP in 3T3-L1 Adipocytes

**Figure 3:**
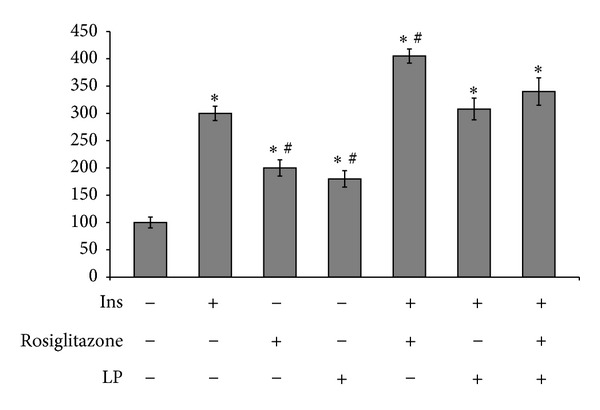
Effects of LP on glucose uptake. Glucose uptake activity in 3T3-L1 adipocytes under insulin-resistant condition in the presence and absence of insulin. The glucose uptake value of untreated cells was set 100, and the others were relative values. Data represented the mean ± SEM (*n* = 5). *P < 0.05 versus control, ^#^ *P* < 0.05 versus insulin only.

Stimulation of cells with 100 nM insulin increased glucose uptake by threefold in the insulin-resistant cells (Figure 3). Incubation of 3T3-L1 adipocytes with rosiglitazone as a positive control for 24 h increased the insulin-stimulated glucose transport activity compared with the absence of rosiglitazone in the control cells. Treatment of LP in basal glucose uptake is comparable to rosiglitazone. LP treatment showed enhancement of insulin-stimulated glucose uptake but not as high as rosiglitazone. However, the glucose uptake in cotreatment was less than that of rosiglitazone monotherapy. It is supposed that the effect of rosiglitazone on glucose uptake was inhibited by LP, and this seems to be related to the effect of PPARgamma transcriptional activity.

## 4. Discussion 

Numerous scientific researches on LP are being carried out, especially for the identification of bioactive phytochemicals that contribute to the pharmacological properties. Most of the phytochemicals that have been identified from the LP extract are phenolic compounds, including phenolic acids and flavonoids as reported [4, 5, 30]. Additionally, in the recent first comprehensive phytochemical study on LP, 19 compounds were isolated from LP [31]. However, isolation of bioactive compounds responsible for the observed activities is necessary for further confirmation. 

 DHT-induced rat PCOS model exhibits both ovarian and metabolic disturbances similar to human PCOS [25]. One of the characteristics is the presence of insulin resistance. We observed that in the DHT-induced PCOS rats, LP treatment resulted in improvement of glucose homeostasis measured using euglycemic-hyperinsulinemic clamp. Meanwhile the 3T3-L1 has been shown to be a useful model when investigating the mechanism of expression of PPARgamma, provided it can differentiate from preadipocytes to adipocytes under certain stimulating conditions [27]. Therefore, in this current study, we determined the expression and protein levels of PPARgamma in the mesenteric adipose tissues and 3T3-L1 cell line in order to further understand the mechanisms involved in the effect of LP on PPARgamma gene and protein expression. 

As shown in the present study, there were higher gene and protein expressions of PPARgamma in the adipose tissues obtained from the DHT-induced PCOS rats treated with LP. Similarly, when insulin-resistant 3T3-L1 cells were treated with LP extract, there was increased insulin-stimulated glucose uptake at the cellular level; both expression and protein levels of PPARgamma were found to be significantly higher compared to the untreated cells. 

Thiazolidinediones (TZD) represent a class of oral hypoglycemic agents that have been shown to improve insulin action and reverse some of the metabolic processes responsible for the development of insulin resistance and, finally, type 2 diabetes in predisposed subjects [32, 33]. As expected, TZD increased significantly compared to control cells. At cellular level, the insulin sensitizing effects of TZDs are mediated through the peroxisome proliferator-activated receptor *γ* (PPAR*γ*) [31] which is highly abundant in adipose tissue and to a lesser extent in skeletal muscle and liver [34, 35]. A representative of TZD, rosiglitazone, is a potent agonist of PPAR*γ* and improves the differentiation of 3T3-L1 cells into adipocytes [12, 13]. Furthermore, adipocyte differentiation leads to the enhanced expression of adipocyte-specific genes, such as GLUT4 and insulin receptor substrate-1 (IRS-1), which are important components of the insulin receptor signal transduction pathway [36, 37].

PPARgamma activation through binding of the synthetic TZDs in type 2 diabetic patients results in a marked improvement in whole-body insulin sensitivity, leading to reduced insulin and glucose plasma levels. At the cellular level, PPARgamma activation has been shown to affect the insulin signaling cascade, through direct modulatory effects on the expression and/or phosphorylation of specific signaling molecules [38]. It is proven that dietary components can bind and activate PPARgamma [17]. However the specificity of the dietary compounds to act as ligands for PPARgamma is still unclear. A metabolic of the parent compound, not the parent compound itself, might be mediating the responces through interactions with PPARgamma. 

Taken together, our studies to date suggest that LP, or more specifically, the compounds present in the aqueous extract, at least partially acts via the PPARgamma pathway, resulting in upregulated PPARgamma. It is not only at the transcriptional level but also at translational level of this nuclear receptor in the adipose tissues, leading to improvement in insulin sensitivity and hence increased uptake of glucose by fat cells. Thus, it may improve the understanding of mechanism of action of LP on insulin sensitivity.
